# Assessment the awareness toward hypertension and diabetes mellitus: Syrian cross sectional study

**DOI:** 10.1186/s12889-023-15666-z

**Published:** 2023-04-28

**Authors:** Sarya Swed, Hidar Alibrahim, Haidara Bohsas, Wael Hafez, Stanisław Surma, Mohammed Amir Rais, Hesham Mohamed Abuelsaoud, Rehab Mohamed Elshazly, Sheikh Shoib, Bisher Sawaf, Amr Farwati, Mohammed Najdat Seijari, Naim Battikh, Soulaf Sleman, Danya Mourad, Komait Jihad Sakkour, Temaa Alklani, Amine Rakab

**Affiliations:** 1 Faculty of medicine, Aleppo University, Aleppo, Syria; 2 NMC Royal Hospital, Khalifa city, abu Dhabi, Khalifa City, Abu Dhabi, UAE; 3 Medical Research Division, Department of Internal Medicine, The National Research Centre, Cairo, Egypt; 4 Faculty of Medical Sciences in Katowice, Medical University of Silesia, Katowice, Poland; 5 Faculty of Medicine of Algiers, University of Algiers, Algiers, Algeria; 6 Nmc royal hospital, Khalifa city, abu Dhabi, UAE; 7 Faculty of Medicine, Ain shams University, Cairo, Egypt; 8 JLNM Hospital, Rainawari, Srinagar India; 9 Directorate of Health Services, J&K, India; 10 Department of Internal Medicine, Hamad Medical Corporation, Doha, Qatar; 11 John H. Stroger, Jr. Hospital of Cook County, Chicago, USA; 12 Faculty of Medicine, Damascus University, Damascus, Syria; 13 Faculty of Medicine, Al-Hawash Private University, Homs, Syria; 14 Faculty of Dentistry, Tishreen University, Lattakia, Syria; 15 Clinical Medicine, Weill Cornell Medical College, Medicine, Qatar

**Keywords:** Hypertension, Diabetes mellitus, Awareness, Syria

## Abstract

**Background:**

Diabetes and arterial hypertension are the two most common types of non-communicable diseases (NCDs) impacting people globally. There is no prior research on the Syrian population’s knowledge and treatment of hypertension and diabetes. It is crucial to investigate how the Syrian public understands and perceives these disorders in order to address the increased incidence and prevalence of hypertension and diabetes. This research intends to assess the level of hypertension and diabetes-related awareness, knowledge, attitude, and practices among Syrian individuals.

**Methods:**

A cross-sectional survey was conducted online between 1 August and 25 August 2022. The questionnaire for the study was developed based on previous research, and the inclusion criteria for the sample were Syrian residents older than 18 who presently live in Syria. The survey consisted four sections: sociodemographics information, WHO STEPS survey instrument on knowledge of and lifestyle determinants for hypertension and diabetes, respondents’ knowledge of and comprehension of hypertension and diabetes, and respondents’ awareness of these disorders.

**Results:**

Among 976 participants, 65.8% were females. the most common causes for hypertension from the perspective of participants were (90.1%) for stress, (87%) High salt consumption, (82.1%) genetics, (78.2%) old age, (78%) obesity (69%) anxiety, and (38.6%) for drug usage. Primary and middle school educational status participants had greater hypertension knowledge (92.3%) than other educational levels. There was a statistical significant difference between the knowledge toward the hypertension and the drinking alcohol, which the nonalcoholic knowledgeable persons were the most common (819 / 976)(P < 0.05). Participants whose lifestyles did not include alcohol use had a higher hypertension knowledge level (90.3%). Participants who do not consume alcohol have shown better hypertension knowledge (90.3%) than those who do (81.9%). Almost age groups have shown good knowledge of diabetes, especially participants aged above 55 (93.8%). However, most individuals have examined blood pressure (82.3%), whereas fewer than half had screened for blood sugar (64.4%). About 82.2% of individuals check their blood pressure frequently, whereas 6.2% monitor their blood sugar. There were significant associations between hypertension knowledge and gender, education, employment, and economic position (P value < 0.05). Men (mean = 8.39, SD = 2.02, P-value < 0.05) have a higher hypertension knowledge than females, and knowledge of hypertension among participants was shown to be higher among those in good income status than other economic levels (mean = 8.34, SD = 1.98). Age, gender, education, employment, and marital status were all associated with diabetes knowledge. Participants between the ages of 40 and 55 showed better knowledge of diabetes compared to other age groups (mean = 11.32, SD = 2.54); also, men demonstrated greater knowledge of diabetes than females (mean = 10.76, SD = 2.79).

**Conclusion:**

We indicated that the Syrian population has a good to moderate understanding of hypertension and diabetes. However, there is still a shortage of standardized, regular screening practices. Since individuals remain involved in unhealthy lifestyle habits, it is vital to provide accurate information about hypertension and diabetes to encourage them to make healthy changes.

## Background

No communicable diseases (NCDs), including cardiovascular disease, cancer, diabetes, and chronic respiratory disorders, are conditions that are caused by a mix of hereditary, physiological, environmental, and behavioral variables. According to WHO, the NCD burden worldwide is predicted to rise by 17% over the next ten year [1]. NCDs are the major cause of mortality around the globe, contributing to 71% of all deaths each year [2], which the risk of NCDs is increased by various modifiable behaviors, including smoking, physical inactivity, unhealthy eating habits, and alcohol abuse. Over 30 million NCD deaths (nearly 80% of all NCD mortality) annually occur in low- and middle-income countries [3]. Although these diseases are increasing globally, low- and middle-income countries and their populations are the most severely afflicted because there is no adequate healthcare environment, especially for the elderly and patients with refractory chronic diseases [4]. Diabetes mellitus and hypertension are the most prevalent diseases in low- and middle-income countries, with two-thirds of all cancer mortality, 90% of COPD deaths, and more than 80% of cardiovascular and diabetes deaths occurring in low-income countries [5]. The presence of both hypertension and diabetes increases the risk of cardiovascular disease (CVD) and the chance of death, raising the risk of nephropathy and retinopathy. According to global statistical reports, 422 million individuals had diabetes at the end of 2016, compared to 1.13 billion people who had hypertension [6]. In addition, American Heart Association data reported that at least 68% of diabetic patients over the age of 65 would die due to cardiovascular illness [7], and the number of individuals with diabetes is expected to rise from 171 million in 2000 to 300 million by 2025 and 366 million by 2030; most of these numerical increases will occur in emerging nations [8]. In Syria, as a highly low-income country, diabetes incidence has almost tripled since 1980, totaling 11.9% in 2016 [9]. Over the course of the last several decades, infectious diseases have given way to non-communicable diseases as the primary public health concern in Syria. [9], before the Syrian crisis, NCDs (mostly cancer, diabetes, and CVDs) were responsible for 77% of all deaths in the country [10, 11]. The prevalence, knowledge, treatment, and management of hypertension vary globally and regionally, according to several worldwide research. In certain countries, the lack of suitable facilities for effective hypertension treatment is an issue for optimal blood pressure control. Al-Hadeethi et al. revealed that many basic health centres in Jordan lack the equipment necessary to provide good hypertension management, whereas Ghareeb et al. had previously shown that the mercury sphygmomanometers in Egypt were not well maintained [12, 13]. According to cross-sectional research conducted in Saudi Arabia, only 37% of hypertensive patients had their blood pressure under control, and only 45% of those who were aware of their condition received treatment [14]. Despite the need for early hypertension prevention and detection, there is evidence to indicate that illness knowledge and blood pressure management are lacking in Syria. Although individual studies on adults in the Syria, there has been no comprehensive research on hypertension prevalence, knowledge, management, and control. It is crucial to evaluate how the Syrian population understands and perceives these disorders in order to address the growing incidence and prevalence of hypertension and diabetes in Syria. Due to the absence of prior observational studies that assess the understanding of the general community toward the characteristics of these illnesses, including risk factors, preventative measures, and appropriate control, it is crucial to evaluate how the Syrian population recognizes and perceives these disorders. As a result, the purpose of this research was to determine the level of awareness, knowledge, attitude, and practices that adults have regarding hypertension and diabetes.

## Methods

### Study destine

From May 5 to June 10, we performed a cross-sectional survey in Syria to assess the degree of knowledge, awareness, and management of hypertension and diabetes among Syrian adults. Syrian citizens aged 18 and above from all Syrian governorates were eligible to participate in the survey. For data collection, we created an online survey using a google form website. We sent it to participants via social media applications such as Facebook, WhatsApp, telegram, and email. A supervising investigator who functioned as the data collection’s mentor was given access to a Google form to guarantee that no fabricated data were included. All participants were informed of the study’s goals, the researchers’ identities, their right to privacy and data protection, their ability to decline participation, and that only fully entered data would be analyzed. This questionnaire was created using data from the World Health Organization (WHO) [15]. and cross-sectional research in Nigeria [16]. The translator expert translated the questionnaire from English to Arabic to confirm that the questions were suitable for the Syrian language.

### Sample size calculation

A standardized tool for sample size calculation of” Survey Monkey” was used to compute the sample size (https://www.surveymonkey.com/mp/sample-size-calculator/). We conducted a statistical power analysis to determine the appropriate sample size using the following parameters: a population percentage of 50%, a margin of error of 0.05, and a confidence level of 95%, according to the latest United Nations data [17]. The Syrian population was estimated at 18,410,965 million people in 2022, so the acceptable sample size was 385. The survey was sent to 997 participants; 7 refused to complete the survey, 14 did not meet the inclusion criteria, and the final sample size was 976 giving a response rate of 97.8%.

### Measures

The questionnaire was split into four main components, which are as follows: (i) personal information about the respondents; (ii) information adapted from the WHO STEPS survey instrument on knowledge of and lifestyle variables for hypertension and diabetes [15]. (iii) The respondents’ knowledge of and understanding of hypertension and diabetes; (iv) the respondents’ awareness of both these diseases. Sixteen items from the STARR County Diabetes Test were modified and adapted from validated surveys on knowledge of hypertension and diabetes Twelve questions were selected from the Hypertension Knowledge Test (HKT) [18]. For this research, a mean knowledge score of at least 6.0 and 8.0 is the highest possible score for hypertension and diabetes, respectively. Scores above these indicate an adequate understanding of the two criteria, while lower scores indicate poor knowledge.

A score of 1 was given for each correct answer, and a score of 0 was given for incorrect and “don’t know” replies for each question on the modified STARR County Diabetes test and the HKT tools. Consequently, each responder could only get a maximum score of 16 and 12, respectively. The mean knowledge ratings for diabetes and hypertension were further divided into “good” and “poor” categories. For knowledge of hypertension, mean scores of eight and above were classified as “good” levels, while scores below eight were classified as “poor” levels. While a mean score of at least six was considered a “good” level of knowledge for diabetes, a score below six was considered “poor” knowledge. The participants who said they measured their blood pressure or blood glucose (BG) level at least once a year were classified as regular checkers, whereas those who said otherwise were classified as non-regular checkers.

### Pilot study

We distributed the questionnaire to 25 randomly selected members of the public to demonstrate its validity and intelligibility. In response, we revised the survey. To assess the validity of the survey, we conducted a pilot test with 25 participants. After performing a pilot study, we distributed the questionnaire and confirmed its acceptable levels of internal consistency (Cronbach’s alpha for the diabetes mellitus and hypertension awareness tools were 0.69 and 0.6, respectively).

### Ethical consideration

The Syrian Ethical Society for Scientific Research awarded the ethical approval. Aleppo University also provided ethical approval (SA-254B). Participants were given a link to the Online Google survey, and on the first page of the survey, they were asked to indicate whether or not they agreed to participate. Afterward, participants are directed to the next page containing significant research information. Complete the questionnaire within five to twelve minutes. Each answer was stored in a secure online database.

### Statistical analysis

Statistical package for the social sciences (SPSS) V.20.0 was used to analyze the data. Means and standard deviation (SD) were used to summarize quantitative data, whereas frequencies and percentages were used to summarize qualitative data. The Shapiro–Wilk test was performed to examine the distribution of the data. Then chi-square (χ2) test analyzed the associations between variables; a p-value of 0.05 was deemed statistically significant. In addition, we performed binary logistic regression to analyze the possibility of getting an adequate level of the knowledge toward hypertension and diabetes mellitus (dependent variables) depending on the sociodemographic variables (Independent variables).

## Results

### Participants’ socioeconomic characteristics

The survey was sent to 997 participants; 7 refused to complete the survey, 14 did not meet the inclusion criteria, and the final sample size was 976, so the response rate was 97%. There were (n = 642, 65.8%) females, and (n = 334, 34.2%) males. The majority of participant were aged between 18 and 25 (n = 705, 72%). Most participants have a university educational status (n = 889, 92.1%), and only (n = 188, 19.3%) were employed. 74.7% participants were city residents, and the majority were singles (n = 749, 76.7%). 26.7% of the participant have low economic status. Table 1.

**Table 1:** Participants’ socioeconomic characteristics:

Variable	Categories	n (%)
**Gender**	**Female**	642 (65.8)
	**Male**	334 (34.2)
**Age groups**	**18–25**	705 (72)
	**25–40**	163 (16.7)
	**40–55**	76 (7.8)
	**55 ≤**	32 (3.3)
**Level of education**	**illiterate**	2 (0.2)
	**Primary school**	13 (1.3)
	**Middle school**	13 (1.3)
	**secondary school**	49 (5.1)
	**University**	899 (92.1)
**Employment status**	**Unemployed**	788 (80.7)
	**Employed**	188 (19.3)
**State of residence**	**City**	729 (74.7)
	**Countryside**	247 (25.3)
**Marital status**	**Single**	749 (76.7)
	**Married**	208 (21.3)
	**Widowed**	10 (1)
	**Divorced**	9 (0.9)
**Monthly income**	**Excellent**	47 (4.8)
	**Good**	261 (26.7)
	**Middle**	410 (42.1)
	**Low**	258 (26.4)

### Hypertension and diabetes awareness and lifestyle modification

Most participants have previously heard about hypertension and diabetes (n = 957, 98.1%), (970, 99.4%), respectively. Only (7.4%) of participants were alcohol consumers, and most alcohol consumers were consuming alcohol less than once a week per month (n = 69, 93.2%). One hundred and eighty-six participants were smokers. Most participants added salt to their meals (85.8%), and more than half exercised at least five minutes weekly (69.1%). Over half of the participants consumed a small quantity of fruit (0 to 3 days per week) (n = 556, 57%), whereas (66.4%) of the participants consumed a sufficient amount of vegetables (4 to 7 days per week). Most of the participant have a family history of hypertension and diabetes (n = 641), (n = 586), respectively. Table 2.

**Table 2:** Hypertension and diabetes awareness and lifestyle modification:

Variable					Categories				n (%)
**Heard about hypertension**			**(N = 976)**		**No**				19 (1.9)
					**Yes**				957 (98.1)
**Heard about diabetes**			**(N = 976)**		**No**				6 (0.6)
					**Yes**				970 (99.4)
**Do you currently take alcohol?**			**(N = 976)**		**No**				904 (92.6)
					**Yes**				72 (7.4)
**Frequency of alcohol consumption among current alcoholics**			**(N = 74)**		**Occasionally (less than 1 week per month)**				69 (93.2)
					**Frequently (daily/weekly)**				5 (6.8)
**Do you currently smoke?**			**(N = 976)**		**No**				790 (80.9)
					**Yes**				186 (19.1)
**Frequency of Smoking among current smokers**			**(N = 210)**		**Occasionally (less than 1 week per month)**				84 (40)
					**Frequently (daily/weekly)**				126 (60)
**Do you add salt to your food on the table?**			**(N = 976)**		**No**				139 (14.2)
					**Yes**				837 (85.8)
**Engage in a form of exercise (at least 5 min per week)**			**(N = 976)**		**No**				302 (30.9)
					**Yes**				674 (69.1)
**Weekly fruits consumption**			**(N = 976)**		**0–3 days**				556 (57)
					**4–7 days**				620 (43)
**Weekly vegetables consumption**			**(N = 976)**		**0–3 days**				328 (33.6)
					**4–7 days**				648 (66.4)
**Family history of hypertension**			**(N = 976)**		**No**				335 (34.3)
					**Yes**				641 (65.7)
**Family history of diabetes**			**(N = 976)**		**No**				390 (40)
					**Yes**				586 (60)
**Variable**	**Hypertension knowledge**					**Diabetes knowledge**			
	**Good (%)**	**Poor (%)**		**Χ2 (P value)**		**Good (%)**	**Poor (%)**	**Χ2 (P value)**	
	**878(90%)**	**98(10%)**				**878(87%)**	**98(13%)**		
**Age (years)**				0.72 (0.869)				8.57 (0.036)	
18–25	635 (90.1)	70 (9.9)				602 (85.4)	103 (14.6)		
25–40	145 (89)	18 (11)				149 (91.4)	14 (8.6)		
40–55	68 (89.5)	8 (10.5)				71 (93.4)	5 (6.6)		
55 and above	30 (93.7)	2 (6.3)				30 (93.8)	2 (6.2)		
**Gender**				0.32 (0.569)				0.24 (0.622)	
Male	303 (90.7)	31 (9.3)				294 (88)	40 (12)		
Female	575 (89.6)	67 (10.4)				558 (86.9)	84 (13.1)		
**State of residence**				1.38 (0.240)				1.04 (0.307)	
City	651 (89.3)	78 (10.7)				641 (87.9)	88 (12.1)		
Countryside	227 (91.9)	20 (8.1)				211 (85.4)	36 (14.6)		
**Highest level of education**				12.19 (0.016)				5.04 (0.283)	
illiterate	2 (100)	0 (0)				2 (100)	0 (0)		
Primary school	12 (92.3)	1 (7.7)				12 (92.3)	1 (7.7)		
Middle school	12 (92.3)	1 (7.7)				11 (84.6)	2 (15.4)		
secondary school	37 (75.5)	12 (14.5)				38 (77.6)	11 (22.4)		
University	815 (90.7)	84 (9.3)				789 (87.8)	110 (12.2)		
**Employment Status**				0.60 (0.437)				1.42 (0.234)	
Unemployed	706 (89.6)	82 (10.4)				683 (86.7)	105 (13.3)		
Employed	172 (91.5)	16 (8.5)				169 (89.9)	19 (10.1)		
**Currently take alcohol**				5.53 (0.019)				2.01 (0.157)	
Yes	59 (81.9)	13 (18.1)				59 (81.9)	13 (18.1)		
NO	819 (90.6)	85 (9.4)				793 (87.7)	111 (12.3)		
**Currently smoke**				0.03 (0.855)				0.11 (0.738)	
Yes	168 (90.3)	18 (9.7)				161 (86.6)	25 (13.4)		
NO	710 (89.9)	80 (10.1)				691 (87.5)	99 (12.5)		
**Physically active**				3.13 (0.077)				1.37 (0.242)	
Yes	614 (91.1)	60 (8.9)				594 (88.1)	80 (11.9)		
NO	264 (87.4)	38 (12.6)				258 (85.4)	44 (14.6)		
**Adds salt to food on the table**				0.001(0.990)				1.43 (0.233)	
Yes	753 (90)	84 (10)				735 (87.8)	102 (12.2)		
NO	125 (89.9)	14 (10.1)				117 (84.2)	22 (15.8)		
**Family history of hypertension**				3.52 (0.061)				4.46 (0.035)	
Yes	585 (91.3)	56 (8.7)				570 (88.9)	71 (11.1)		
NO	293 (87.5)	42 (12.5)				282 (84.2)	53 (15.8)		
**Family history of diabetes**				6.63 (0.010)				5.97 (0.015)	
Yes	539 (92)	47 (8)				524 (89.4)	62 (10.6)		
NO	339 (66.9)	51 (13.1)				328 (84.1)	62 (15.9)		

### Knowledge of the causes, risk factors, symptoms, consequences, prevention, and control measures of hypertension and diabetes

#### Causes

The hypertension causes reported by the participants were: (n = 898, 90.1%) stress, (n = 772, 78.2%) older age, (n = 681, 69%) anxiety, and (n = 381, 38.6%) drug abuse.

It was determined that genetics (n = 915, 92.7%), insulin resistance (n = 774, 78.4%), stress (n = 490, 49.6%), and insulin resistance (n = 526, 53.3%) are all diabetes causes.

#### Signs & symptoms

The most common signs of high blood pressure that the respondents indicated were headaches (n = 852, 86.3%), palpitations (n = 695, 70.4%), and dizziness (n = 620, 62.8%). Hypertension was reported to be asymptomatic by only 4.1% (n = 40).

Frequent urine (n = 930, 94.2%) and non-healing wounds (n = 790, 80.4%) are indications of diabetes, according to participant reports.

#### Risk factors

Excessive salt consumption (n = 859, 87%), heredity (n = 810, 82.1%), obesity (n = 769, 78%), and high fat intake (n = 717, 72.6%) have all been identified to be hypertension risk factors by participants.

Diabetes risk factors recognized were heredity (n = 882, 89.4%), obesity (n = 759, 76.9%), and excessive fat consumption (n = 511, 51.8). Only (n = 340, 34.4%) and (n = 325, 33%) of participants considered excessive alcohol use and smoking as risk factors for diabetes, respectively.

#### Complications

In terms of hypertension complications, participant response was (n = 748, 75.8%) for foot ulcer, (n = 730, 74%) for vision loss, and (n = 638, 64.6%) for chronic kidney disease.

Responses from participants for hypertension-related complications were (n = 737, 74.7%) for stroke, (n = 643, 65.1%) for heart failure, and (n = 625, 63.3%) for death.

#### Prevention methods

Reduced salt consumption (n = 912, 92.4%), regular exercise (n = 861, 87.2%), and reduced anxiety (n = 839, 85%) were all cited as effective strategies for preventing hypertension.

Avoiding sugary foods (n = 857, 86.8%), taking antihypertensive medication (n = 842, 85.3%), and maintaining a regular exercise routine (n = 780, 79%) were all mentioned by participants as practical means of preventing diabetes (Table 3).

**Table 3:** Knowledge of the causes, risk factors, symptoms, consequences, prevention, and control measures of hypertension and diabetes:

Item	Hypertension n (%)	Item	Diabetes n (%)
Causes		Causes	
**Stress**	898 (90.1)	**Insulin resistance**	774 (78.4)
**Old age**	772 (78.2)	**Drug abuse**	213 (32.6)
**Drug abuse**	381 (38.6)	**Old age**	526 (53.3)
**Anxiety**	681 (69)	**Stress**	490 (49.6)
**Unknown**	47 (4.8)	**Hereditary**	915 (92.7)
		**Excess Sugar Intake**	66.8 (67.7)
**Risk factors**		**Risk factors**	
**Heredity**	810 (82.1)	**Heredity**	882 (89.4)
**Smoking**	696 (70.5)	**Smoking**	325 (33)
**Obesity**	769 (78)	**Obesity**	759 (76.9)
**High fat intake**	717 (72.6)	**High fat intake**	511 (51.8)
**Excess alcohol** **intake**	518 (52.5)	**Excess alcohol** **intake**	340 (34.4)
**High salt intake**	859 (87)	**High salt intake**	99 (10)
**Don’t Know**	35 (3.5)	**Don’t Know**	51 (5.2)
**Symptoms**		**Symptoms**	
**Headache**	852 (86.3)	**Frequent urination**	930 (94.2)
**Dizziness**	620 (62.8)	**Non-healing wounds**	794 (80.4)
**Palpitations**	695 (70.4)	**No signs**	28 (2.8)
**Poor vision**	526 (53.3)		
**Don’t know**	48 (4.9)		
**No signs**	40 (4.1)		
**Complications**		**Complications**	
**Stroke**	737 (74.7)	**Stroke**	383 (38.8)
**Death**	625 (63.3)	**Heart failure**	343 (34.7)
**Heart failure**	643 (65.1)	**Chronic kidney disease**	638 (64.6)
**Loss of sight**	456 (46.2)	**Loss of sight**	730 (74)
**Chronic kidney disease**	477 (48.3)	**Foot ulcer**	748 (75.8)
**Don’t know**	112 (11.3)	**Death**	471 (47.7)
		**Don’t know**	83 (8.4)
**Prevention / control**		**Prevention / control**	
**Minimizing salt intake**	912 (92.4)	**Minimizing salt intake**	84 (8.5)
**Reducing intake of fatty foods**	743 (75.3)	**Reducing intake of fatty foods**	534 (54.1)
**Avoiding excessive intake of alcohol**	606 (61.4)	**Avoiding excessive intake of alcohol**	380 (38.5)
**Avoiding smoking**	734 (74.4)	**Avoiding smoking**	327 (33.1)
**Regular exercise**	861 (87.2)	**Regular exercise**	780 (79)
**Taking Antihypertensive (drugs)**	780 (79)	**Taking Antihypertensive (drugs)**	842 (85.3)
**Avoiding anxiety**	839 (85)	**Avoiding Excess sugar**	857 (86.8)
**Prayer**	448 (45.4)	**Don’t know**	30 (3)
**Don’t know**	18 (1.8)		

### Knowledge of hypertension and diabetes in relation to socioeconomic factors and lifestyle practices

#### Hypertension knowledge

Participants’ overall hypertension awareness was high (n = 878, 89.9%).

We found that 92.3% of elementary and middle school students being more knowledgeable about hypertension than other educational levels(P-vale < 0.05). It was also determined that those who do not consume alcohol had better hypertension knowledge (90.3%)(P-vale < 0.05).

#### Diabetes knowledge

Among all participants (n = 852, 87.2%) had a good knowledge of diabetes. The participants above the age of 55 showed more diabetes awareness than other(P-vale < 0.05). Knowledge of diabetes is higher among those with a family history of hypertension (88.9%) and diabetes (89.4%)(P-vale < 0.05) (Table 4).

**Table 4:** Knowledge of hypertension and diabetes status and associated treatment methods

Hypertension				Diabetes			
**Variable**			**n (%)**	**Variable**			**n (%)**
**Ever been screened for high blood pressure**	(n = 976)	No	173(17.7)	**Ever been screened for high blood sugar?**	(n = 976)	No	523 (53.6)
		Yes	803 (82.3)			Yes	453 (46.4)
**Last blood pressure check**	(n = 837)	0–3 months ago	429 (51.2)	**Last blood sugar check**	(n = 586)	0–3 months ago	194 (33.1)
		4–6 months ago	87 (10.4)			4–6 months ago	85 (14.5)
		> 6 months ago	321 (38.4)			> 6 months ago	307 (52.4)
**Frequency of blood pressure check**	(n = 976)	At least once in a month	188 (19.3)	**Frequency of blood sugar check**	(n = 642)	At least once in a month	75 (11.7)
		At least once in a year	166 (17)			At least once in a year	171 (26.6)
		Occasionally (*when sick*)	622 (63.7)			Occasionally (*when sick*)	396 (61.7)
**Ever been diagnosed of high blood pressure?**	(n = 976)	No	888 (91)	**Ever been diagnosed of high blood sugar?**	(n = 976)	No	915 (93.7)
		Yes	88 (9)			Yes	61 (6.3)
**Were you prescribed medication for your condition?**	(n = 675)	No	612 (90.7)	**Were you prescribed medication for your condition**	(n = 623)	No	575 (92.3)
		Yes	63 (9.3)			Yes	48 (7.7)
**Do you take you medication regularly?**	(n = 552)	No	428 (77.5)	**Do you take you medication regularly?**	(n = 525)	No	432 (82.2)
		Yes	124 (22.5)			Yes	93 (17.7)
**Ever been screened for high blood sugar?**	(n = 976)	No	523 (53.6)				
		Yes	453 (46.4)				

### Knowledge of hypertension and diabetes status and associated treatment methods

#### Blood pressure screening

Most of the involved individuals (n = 803, 82.3%) had at least once undergone a blood pressure screening. In the last three months, about half of the individuals (n = 429, 51.2%) had checked their blood pressure. 91% of the participants in this study had been diagnosed with hypertension at some point in their lives.

#### Blood sugar screening

More than half of the individuals (n = 453, 64.4%) had previously undergone a blood sugar screening. Approximately (33.1%) of participants had attended a blood sugar screening during the last three months. Only 61 (6.3%) of the individuals had been previously diagnosed with high blood sugar. 

### Regular monitoring of blood pressure and blood sugar among respondents

#### Check blood pressure regularly

Regular blood pressure monitoring was performed by (82.2%) of participants. All respondents aged older than 55 frequently monitor their blood pressure. University-educated participants check their blood pressure regularly (83.5%) more than other education subgroups (P-vale < 0.05). Most people with excellent or poor hypertension consistently monitor their blood pressure (83.5% and 71.4%, respectively;P-vale < 0.05).

#### Check blood sugar regularly

About 6.2% of participants routinely monitored their blood sugar levels. Frequent blood sugar monitoring was significantly associated with age, place of residency, history of hypertension, family history of diabetes, knowledge of hypertension, and knowledge of diabetes (P value < 0.05). Most people aged 40 to 55 (85.5%) check their blood sugar more often than other age groups, as do city residents (48.6%) more frequently than residents of rural regions. Participants with good knowledge of diabetes (48.1%) check their blood sugar regularly more than those who do not. Participants with good diabetes knowledge (48.1%) check their blood sugar levels more frequently than those without. (Table 5)

**Table 5:** Regular monitoring of blood pressure and blood sugar among respondents:

Variable	Check BP regularly? (n = 976)			Check BS regularly? (n = 976)		
	No (%) (n = 173)	Yes (%) (n = 803)	Χ2 (P value)	No (%) (n = 915)	Yes (%) (n = 61)	Χ2 (P value)
**Age (years)**			14.41(0.002)			84.3 (< 0.001)
18–25	131 (18.6)	574 (81.4)		434 (61.6)	271 (38.4)	
25–40	36 (22.1)	127 (77.9)		71 (43.6)	92 (56.4)	
40–55	6 (7.9)	70 (92.1)		11 (14.5)	65 (85.5)	
55 and above	0 (0)	32 (100)		7 (21.9)	25 (78.1)	
**Gender**			2.64 (0.104)			1.47 (0.225)
Male	50 (15)	284 (85)		170 (50.9)	164 (49.1)	
Female	123 (19.2)	519 (80.8)		353 (55)	289 (45)	
**State of residence**			0.38 (0.535)			5.33 (0.021)
City	126 (17.3)	603 (82.7)		375 (51.4)	354 (48.6)	
Countryside	47 (19)	200 (81)		148 (59.9)	99 (40.1)	
**Highest level of education**			14.03(0.007)			6.49 (0.165)
illiterate	1 (50)	1 (50)		2 (100)	0 (0)	
Primary school	5 (38.5)	8 (61.5)		5 (38.5)	8 (61.5)	
Middle school	5 (38.5)	8 (61.5)		10 (76.9)	3 (23.1)	
secondary school	14 (28.6)	35 (71.4)		29 (59.2)	20 (40.8)	
University	148 (16.5)	751 (83.5)		477 (53.1)	422 (46.9)	
**Average monthly income**			4.78 (0.188)			3.7 (0.296)
Excellent	11 (23.4)	36 (76.6)		19 (40.4)	28 (59.6)	
Good	42 (16.1)	219 (83.9)		142 (54.4)	119 (45.6)	
Middle	65 (15.9)	345 (84.1)		219 (53.4)	191 (46.6)	
Low	55 (21.3)	203 (78.7)		143 (55.4)	115 (44.6)	
**Currently consume alcohol**			1.46 (0.228)			1.26 (0.261)
No	164 (18.1)	740 (81.9)		489 (54.1)	415 (45.9)	
Yes	9 (12.5)	63 (87.5)		34 (47.2)	38 (52.8)	
**Currently smoke**			0.04 (0.836)			1.57 (0.210)
No	141 (17.8)	649 (82.2)		431 (54.6)	359 (45.4)	
Yes	32 (17.2)	154 (82.8)		92 (49.5)	94 (50.5)	
**Physically active**			2.95 (0.086)			0.99 (0.319)
No	63 (20.9)	239 (79.1)		169 (56)	133 (44)	
Yes	110 (16.3)	564 (83.7)		354 (52.5)	320 (47.5)	
**Family history of hypertension**			0.41 (0.523)			3.84 (0.050)
No	63 (18.8)	272 (81.2)		194 (57.9)	141 (42.1)	
Yes	110 (17.2)	531 (82.8)		329 (51.3)	312 (48.7)	
**Family history of diabetes**			1.1 (0.294)			11.62 (0.001)
No	63 (16.2)	327 (83.8)		235 (60.3)	155 (39.7)	
Yes	110 (18.8)	476 (81.2)		288 (49.1)	289 (50.9)	
**Hypertension knowledge**			8.79 (0.003)			5.01 (0.025)
Poor	28 (28.6)	70 (71.4)		63 (64.3)	35 (35.7)	
Good	145 (16.5)	733 (83.5)		460 (52.4)	418 (47.6)	
**Diabetes knowledge**			2.29 (0.130)			7.87 (0.005)
Poor	28 (22.6)	96 (77.4)		81 (65.3)	43 (34.7)	
Good	145 (17)	707 (83)		442 (51.9)	410 (48.1)	

### The difference in knowledge of hypertension and diabetes in relation to demographic factors

#### Association between variables and hypertension knowledge

Four out of seven factors were statistically significant predictors of hypertension knowledge, including gender, educational status, occupational status, and economic level (P-value < 0.05). Men have a higher hypertension knowledge than females (mean = 8.39, SD = 2.02), and university-educated participants had a higher hypertension knowledge than other educational levels (mean = 8.19, SD = 2.07). Knowledge of hypertension among participants was shown to be higher among those in good income status than other economic levels (mean = 8.34, SD = 1.98).

#### Association between variables and diabetes knowledge

Statistics showed that greater diabetes knowledge was linked to 5 of the 7 factors (age, gender, education, occupation, and marital status) (P-value < 0.05). Participants between the ages of 40 and 55 showed better knowledge of diabetes compared to other age groups (mean = 11.32, SD = 2.54); in addition, men demonstrated greater knowledge of diabetes compared to females (mean = 10.76, SD = 2.79). Participants with a university degree showed more diabetes knowledge than other educational levels education (mean = 10.61, SD = 2.76) (Table 6).

**Table 6:** The difference in knowledge of hypertension and diabetes in relation to demographic factors:

Variable	Hypertension knowledge			Diabetes knowledge		
	Mean (SD)	95%CI (lower-upper)	P-value	Mean (SD)	95%CI (lower-upper)	P-value
**Age (years)**			0.512			< 0.001
18–25	8.1 (2.088)	7.95–8.26		10.31 (2.832)	10.1–10.52	
25–40	7.97 (2.153)	7.64–8.3		11.1 (2.512)	10.71–11.49	
40–55	8.13 (2.205)	7.63–8.64		11.32 (2.541)	10.74–11.9	
55 and above	8.56 (1.917)	7.87–9.25		10.97 (2.102)	10.21–11.73	
**Gender**			0.002			0.043
Male	8.39 (2.029)	8.17–8.6		10.76 (2.790)	10.46–11.06	
Female	7.95 (2.125)	7.78–8.11		10.43 (2.741)	10.22–10.64	
**State of residence**			0.832			0.992
City	8.09 (2.113)	7.93–8.24		10.56 (2.747)	10.36–10.76	
Countryside	8.14 (2.073)	7.88–8.4		10.5 (2.807)	10.15–10.85	
**Highest level of education**			< 0.001			0.043
illiterate	7 (1.414)	-5.71–19.7		9.5 (0.707)	3.15–15.85	
Primary school	7 (1.225)	6.26–7.74		10.54 (2.106)	9.27–11.81	
Middle school	7.15 (1.345)	6.34–7.97		9.69 (2.394)	8.25–11.14	
secondary school	6.94 (2.427)	6.24–7.64		9.59 (2.857)	8.77–10.41	
University	8.19 (2.079)	8.06–8.33		10.61 (2.763)	10.43–10.79	
**Employment Status**			0.014			< 0.001
Unemployed	8.03 (2.121)	7.88–8.17		10.38 (2.795)	10.18–10.57	
Employed	8.4 (1.999)	8.12–8.69		11.24 (2.499)	10.88–11.6	
**Marital status**			0.279			0.011
Single	8.15 (2.135)	8–8.31		10.4 (2.850)	10.2–10.6	
Married	7.91 (1.955)	7.64–8.18		10.92 (2.425)	10.59–11.25	
Widowed	8.3 (1.636)	7.13–9.47		12.3 (1.567)	11.18–13.42	
Divorced	7.78 (2.991)	5.48–10.08		11.78 (1.856)	10.35–13.2	
**Monthly income**			0.014			0.882
Excellent	7.72 (2.233)	7.07–8.38		10.6 (3.090)	9.69–11.5	
Good	8.34 (1.987)	8.1–8.59		10.64 (2.675)	10.31–10.96	
Middle	8.16 (2.113)	7.95–8.36		10.53 (2.749)	10.26–10.79	
Low	7.82 (2.144)	7.56–8.08		10.47 (2.815)	10.12–10.81	

## Discussion

Non-communicable diseases (NCDs) are a major health problem. Major NCDs include cancer, cardiovascular disease, chronic respiratory disease, diabetes, and musculoskeletal disorders [19]. The increase in non-communicable diseases is mainly attributable to four major risk factors: smoking, lack of physical activity, excessive alcohol consumption, and poor diet [20]. High blood pressure and diabetes are two frequently associated diseases considered serious conditions. These disorders are often asymptomatic, making the diagnosis relatively hard to establish. Hence, rapid identification is mandatory to prevent major complications [21, 18]. This study aimed to evaluate individuals’ knowledge, awareness, and attitude toward diabetes and hypertension. Findings showed that out of 976 participants, only 19 and 6 had not heard about hypertension and diabetes, corresponding to an excellent knowledge rate among the population of 98.1% and 99.4%. This is strongly concomitant with the finding of a study by Amandi et al. in which 96% and 92% of the respondents were aware of hypertension and diabetes [22, 19], but not concomitant with findings of a study of seven communities in East and West Africa, which showed 43% of respondents with hypertension were not heard about this dieses. Our results reported that the most incriminated cause chosen by responders is stress for hypertension (90.1%) and genetic factors for diabetes (92.7%). Stress as an important risk factor for arterial hypertension was also indicated in other surveys. In our study, alcohol consumption was found to be a well-known factor in cardiovascular diseases, which is similarly found in a Nigerian study by Jennifer et al. [16]. Alcohol intake and smoking among the study participants were found to be low (7.4% and 19.1%) in contrast to salt adding, which has been identified as frequently done (85.5%). Although there was a slight difference in the knowledge of diabetes and hypertension in elderly patients compared with other age groups and in patients with a family history It may be ascribed to the increased focus on health as people age, as well as the increased likelihood of developing HTN and the increased frequency of sickness in older folks., the strongest demographic factor was the education level; findings showed that primary and middle school students had greater hypertension knowledge (92.3%) (P < 0.05). These results are similar to findings found in an Indian study by Sneha et al.; where education was found to be a significant influence factor on diabetes and hypertension knowledge [23], in contrast, Russian study showed that being low-educated was associated with a lower rates of awareness of hypertension [24].

On the other hand, our results demonstrated that men have a higher hypertension and diabetes knowledge than females (mean = 8.39, 10.76, respectively), whereas, the women Czech and Polish women were more knowledgeable about these dieses according to Wentian Lu et al. [25]. Whereas another research conducted in Kazakhstan found that women had much greater rates of diabetes awareness than males [26].

Furthermore, our study shown that individuals with obesity and excess weight had a greater possibility of having an adequate level of knowledge toward hypertension than those with normal BMI; this correlation is supported by research literature and other studies [27, 28].

In compared to low- and middle-income nations, high-income nations had almost double the proportions of awareness (67.0 vs. 37.9%), therapy (55.6 vs. 29.0%), and control among hypertension patients (28.4 vs. 7.7%) [29].

Currently, several studies have shown that hypertension and diabetes mellitus are extremely prevalent in low- and middle-income countries (LMIC) and are considered a leading cause of morbidity and mortality [30]. It is, therefore, essential to highlight several approaches and strategies to maximize knowledge and attitude toward these pathologies and increase awareness. Also, Efforts should be made to incorporate worldwide recommendations in daily practice in order to enhance hypertension control rates in Syria It appears that sensibilization toward diabetes and hypertension is still very much achievable as education on their complications, symptoms, and risk factors can be addressed by most healthcare practitioners, such as general physicians, pharmacists, and dentists in hospitals, private clinics, schools, during awareness events, conferences and scientific meetings and through online social media articles [31]. In addition, generalized free screening events are a strong strategy for early detection, correct management, and easy care of diabetes and hypertension [32]. We found that 63.7% of those who responded checked their blood pressure periodically or if they were unwell, and that 53.6% had never had their blood sugar checked. The low screening and checkup rate is a serious public health concern that must be addressed immediately and adequately by increasing access to cost-free methods of controlling chronic illnesses. In addition, advice for preventing hypertension and type 2 diabetes, such as cutting down on sugar and salt, doing activities that relieve stress, getting regular exercise, not drinking too much alcohol, quitting smoking, and staying away from secondhand smoke, should be urged [33, 34]. The early detection and treatment of these illnesses at the primary care level will benefit from screening asymptomatic individuals for these disorders. Appropriate counselling to enhance adherence to treatment and advise will almost certainly result in improved management of these illnesses. This underscores the need for interventions that concentrate on the phases of the cascade that deal with awareness and treatment, even if changes are required throughout the full hypertension and diabetes care cascade. Effective implementation of the current national NCD programs is urgently required across all Syrian provinces. Fat and excessive alcohol intake and smoking, despite their proven incrimination in diabetes, findings showed they weren’t found to be a strong factor in diabetes cause according to the responders, this false information should also be corrected and spread in awareness and sensibilization events. Should be noted that education in healthy eating habits and a healthy lifestyle is the cornerstone of cardiovascular prevention. Starting such education from an early age significantly reduces the risk of cardiovascular diseases in the future, as demonstrated research group led by Valentin Fuster [35, 36]. In order to maintain a good effect of the conducted health education, it should be repeated periodically [37].

### Limitations and strengths

Despite being a free and straightforward method of data collecting, our research is a cross-sectional study of the present kind, which may not reveal the genuine proper causality and prevalence. Since the poll was sent through a Google Form to social networking sites, it was not accessible to the elderly, individuals living in rural regions, people without access to the internet, or people without email, making it difficult to completely eradicate prejudice. Another limitation of the study is the lack of assessment of the respondents’ knowledge of non-classical risk factors for hypertension and diabetes, such as air pollution, periodontitis. Despite the fact that we identified many restrictions, there are also many benefits, such as the fact that we circulated the questions and collected responses from all Syrian administrations, as well as the fact that we took the consent of each participant before responding on the online survey at the first page, and there was an expert investigator to monitor the safety of the data collection process.

## Conclusion

Our research has shown that the degree of awareness about diabetic mellitus and hypertension is reasonable; nonetheless, this level of knowledge is absolutely needed to be improved among the Syrian population. This is because virtually all statistical reports define a high prevalence of these illnesses, along with a spreading high probability of having these disorders since the Syrian population is posed of many risk factors. The local recommendations for the prevention and treatment of these illnesses need to be updated so that they match the most recent version of the guidelines. Through collaboration between international health organizations and the Syrian government’s health authority, raising awareness should be seen as a significant first step in finding a solution to this present problem.

## Data Availability

The authors have access to and have saved all the data necessary to support this paper’s conclusion. All data are accessible upon reasonable request from the corresponding author.
